# Creating conditions for a sense of security during the evenings and nights among older persons receiving home health care in ordinary housing: a participatory appreciative action and reflection study

**DOI:** 10.1186/s12877-019-1372-z

**Published:** 2019-12-16

**Authors:** Inger James, Margaretha Norell Pejner, Annica Kihlgren

**Affiliations:** 1 School of Health Sciences, Department of Nursing Sciences, Örebro University, SE-701 82 Örebro, Sweden; 2 School of Health and Welfare, Department of Health and Nursing, Halmstad University, Halmstad, Sweden

**Keywords:** Evenings and nights, Home health care, Older persons, Ordinary housing, Participatory appreciative action and reflection, Sense of security

## Abstract

**Background:**

Today many older persons in ordinary housing receive home health care. It is stipulated that the care is to provide security; however, deficiencies in home health care are reported in many countries. It may be difficult to implement a sense of security among older persons receiving home health care in ordinary housing, especially during the evenings and nights, due to a lack of knowledge.

**Methods:**

This study is part of a larger project with a participatory appreciative action and reflection (PAAR) approach. We invited older persons, relatives, nurse assistants, registered nurses, and their managers to co-create knowledge with us on how conditions for a sense of security can be created during evenings and nights among older persons receiving home health care in ordinary housing. We performed thematic analysis of the data.

**Results:**

Five subthemes were developed that gave structure to two main themes. The first main theme, To confirm the self-image, has the following subthemes: To see the home as a reflection of the person’s identity and To maintain self-determination. The second main theme, To create interaction in a sheltered place, has these subthemes: To undress the power, To create control and lifelines, and To create a good sleeping environment. The two themes interact and are each other’s conditions. The person’s self-image must be confirmed in order to create interaction in a sheltered place and through the interaction, the self-image is confirmed.

**Conclusion:**

Conditions necessary for older persons to have a sense of security are living in a familiar environment, having habits and routines maintained, and having self-determination. Other conditions are equality, the prevention of falls, and an individualized sleeping environment. Older people’s self-determination should be honored, and they should not being excluded from decision-making. We need to ask them if the conditions are sufficient and their sense of security is great enough to allow them to continue living in their ordinary housing.

## Background

This study is about how older persons receiving home health care in ordinary housing can have a sense of security during the evenings and nights. With longer life expectancies, more than half of the increasingly aging population [1] is diagnosed with multiple illnesses that impose major consequences related to increased health care utilization [2]. Even with well-known health problems, many older persons express a desire remain in their residences that do not offer specialized services, in so-called ordinary housing, and receive help from home health care and/or relatives [3–6]. Home health care for older persons involves a give and take of autonomy and dependency [7]. To improve autonomy and independence, older people must be able to make their own decisions, manage their health and daily lives, and maintain social contacts. When they are no longer able, they may engage the use of caregivers as an extension of themselves [7, 8], people they know they can get help from [8].

However, deficiencies in the home health care delivered to those living in ordinary housing are reported in many countries [9, 10]. Deficiencies have been attributed to fragmented home visits [11, 12], the staff’s lack of time [13–15], and visits that are too hasty [12]. The care can focus on “doing,” where daily tasks are performed mechanically without regard to the person’s individual needs [12]. People who are dependent on home health care are not always getting the help they need [16]. Older people have to move to nursing homes because in their ordinary housing they lack a sense of security; furthermore, due to physical or cognitive impairment, they can have difficulty managing routine activities and keeping up with daily life [17]. Their sense of insecurity may be relative to the time of day, with the greatest levels occurring in the evenings and nights. However, there is a lack of knowledge regarding this, as only a few national and international studies have focused on the evenings and nights, and most of these are from nursing homes [18]. The challenges in nursing homes with older people with dementia and perceived sleep disturbances at night are exacerbated by insufficient staffing levels [19]. Older persons often have to adapt to the staff’s working hours, which means they get up early, and those requiring the most assistance spend more than the average 11 h in bed at night and significantly more hours awake in bed [20].

There are challenges even for older people who choose to remain in their ordinary housing with relatives as their informal caregivers providing comprehensive care during the evenings and nights [21, 22]. The relatives describe poor sleep quality and symptoms of fatigue [22]. Older persons with care that includes nightly monitoring checks may simply stay awake, waiting for staff to come [23]. Those who receive help may have unmet needs due to illnesses and medical treatments required at night [24].

The World Health Organization [25] stipulates that older persons in ordinary housing should be able to have a sense of security as well as freedom. The Swedish Social Services Act guarantees that older people will have the opportunity to live independently with dignity and in conditions conducive to a sense of security [26–29]. The individual should be the focus, and help should be given quickly, whether it is during the day or in the middle of the night [23]. Although it is stipulated that the health care system must ensure this sense of security [26], it may be difficult to achieve it [18].

Security has been described in different ways, and Segesten [30, 31] describes it as a sense that no one can mediate, but one for which the conditions can be created. As such, there is a need to take into account the perspectives of older persons receiving home health care in ordinary housing [11], and to ask them what creates a sense of security for them during the evenings and nights. It is also important to listen to those who can create the conditions for a sense of security, that is, relatives, nurse assistants (NAs), registered nurses (RNs), and managers, and to ask them what a sense of security for older persons can entail.

## Methods

### Aim

The aim was to describe the stakeholders’ views about how conditions for a sense of security can be created during evenings and nights among older persons receiving home health care in ordinary housing.

### Design

This study is part of a larger project where we theoretically and methodologically used a participatory appreciative action and reflection (PAAR) design. The stakeholders invited in this study—older persons receiving home health care in ordinary housing, relatives to persons receiving home health care, NAs, RNs, and their managers working in home health care—were invited to co-create knowledge with us regarding how a sense of security can be created for the older persons during evenings and nights. Our use of sense of security is in accordance with Segesten’s definition, where one can create the conditions for someone to have a sense of security, which is based on the individual’s perception of it [30, 31].

The design is underpinned by the view of the stakeholders as narrators of their own stories and interpreters of their own lives and work. This research has its roots in participation, action, and reflection by the stakeholders. It consists of repeated cycles of collecting data, taking it back to the stakeholders, analyzing it, and considering it in reflection, as well as collecting new data. This is done with the aim of learning from the stakeholders’ experience and knowledge [32, 33]. An appreciative perspective was applied, and we tried to identify what has worked well to create conditions for a sense of security for older people in practice, as well as solutions to the obstacles [34].

### Sample

#### Stakeholders and setting

Since the home health care service in the municipality is rather extensive, we could not invite all of the possible stakeholders. Therefore we used a purposeful sampling to gain in-depth insight into the phenomenon [35]. We selected individuals 65 years of age and over, living in ordinary housing. We included three different home health care units to achieve variation in urban and rural areas receiving home health care during the evenings and nights within a larger municipality in Sweden. The staff who worked on evening and night shifts from the three different home health care units selected also participated in the study.

Oral and written information regarding the study was given by the respective unit managers to the older persons in their homes and to the staff at staff meetings. The researcher contacted the older people after they had signed the written consent. Altogether, 18 older individuals participated, 14 women and 4 men between the ages of 79 and 97 years. The assistance and support the older persons needed varied. Some had only monitoring visits during evenings and nights, while others had extensive health care requirements. Since some of the older persons did not have relatives, or the relatives did not wish to participate, relatives of persons receiving home care were recruited through an agency that provides support to relatives in the municipality. Five relatives, all women aged 67–82 years, agreed to participate. Among the 40 NAs who agreed to participate were 38 females and two males aged 21–68 years who had worked in the profession between six months and 40 years. The one male and nine female RNs who participated were aged 36–70 years and had worked in their profession 8–48 years. The four unit managers of the evening shifts and the three managers of the night shifts in the municipality were invited, and all agreed to participate.

### Data collection

To learn from all of the stakeholders’ experiences and knowledge, data were collected through individual interviews, focus group discussions, observations, and informal conversations. In line with action research, the interviews, focus group discussions, and informal conversations were conducted in the form of reflective conversations. We adapted to the stakeholders’ perspectives of the conditions required for a sense of security. During the data collection, comments and ideas generated new discussions; for example, if the older persons talked about how conditions in the home gave them a sense of security, the other stakeholders might be asked about that. The first author (IJ) made the data collection and compiled the data. A reference group was formed with representatives from all of the stakeholders who participated in the study. The reference group consisted of three managers from evening and night shifts, one unit manager, one RN, one relative, three NAs, and two older persons, along with two researchers. The group analyzed and reflected on all of the compiled data, which were then presented to the stakeholders for a second and third time to further reflect on the conditions necessary for a sense of security. In this way, a consensus was reached. We strived to create openness in an open atmosphere where no one would feel bound or obligated to the organization. In this “free space,” the stakeholders and the researcher together reflected and learned how to create conditions for a sense of security [36].

#### Interviews and focus group discussions

The interviews were conducted in the older persons’ homes according to their preference and at the times they chose. The older people were interviewed one to three times, which resulted in 25 interviews that were conducted in the afternoons or evenings. Appreciative intent and confirmation through interaction was sought to co-create knowledge in each interview. Open, reflective questions such as “What creates conditions for a sense of security or insecurity during the evenings and nights for you?” were asked. Reflections on the possibilities and obstacles related to a sense of security were raised. Follow-up questions included “How can these possibilities be achieved?” “What obstacles to your sense of security may be found during evenings and nights, and how can these obstacles be resolved?” The interviews were transcribed verbatim by a secretary and were documented chronologically and compiled on a continuing basis. In a next step, the compilations were taken back to the older persons, who, together with the researcher, provided their analyses and reflections on the content of what had been said regarding a sense of security or insecurity during the evenings and nights. Relatives participated in two focus group discussions, which were conducted at the center operated by the agency that supports relatives. An additional researcher attended to take notes and ask additional follow-up questions. Managers, RNs, and NAs participated in individual interviews and focus group discussions that took place at their workplaces. After the transcripts were compiled, they were taken back two or three times to the stakeholders for their analyses and reflections on the content.

#### Observations and informal conversations

During the repeated observations, the researcher followed the RNs and the NAs while they were working. There were 183 h of observation performed between 16:00 in the afternoon and 07:00 in the morning (113 h during the evening shifts and 70 h during night shifts). The informal conversations and observations between the researcher, the nursing staff (NAs and RNs), and the older persons were tape-recorded or documented in field notes. The conversations were not a result of any planned questions, but rather discussions with reflective responses. The informal conversations were conducted between the caregiving episodes. They could be encounters between the researcher, the older people, and the nursing staff when, for example, the conversation began with the topic of what creates a sense of security or insecurity. The field notes and transcribed tape-recordings were compiled and taken back to the stakeholders in the same way as the interviews and discussions were.

### Data analysis

**Fig. 1:**
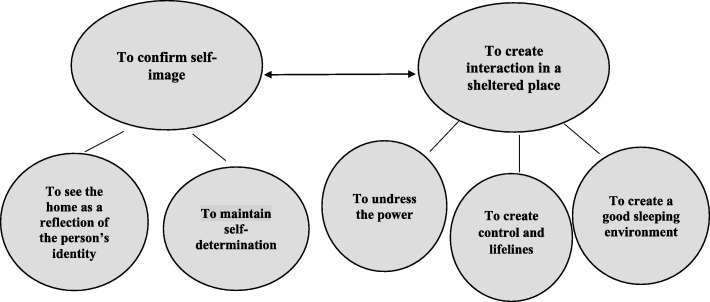
Creating conditions for a sense of security. Relationships between main themes and subthemes

After the preliminary analysis was conducted with the stakeholders and a consensus was reached regarding the content, the researchers chose to use thematic inductive analysis because “through its theoretical freedom, thematic analysis provides a flexible and useful research tool, which can potentially provide a rich and detailed, yet complex, account of data..”(37, p. 78). The thematic inductive analysis is data-driven from a bottom-up perspective. The researchers performed thematic analysis of the data in several steps [37]. We posed this question to the data: *How do the stakeholders describe the opportunities and obstacles for creating conditions conducive to a sense of security for older persons?* In the first step we became acquainted with the data by reading and rereading the compilations. Second, we identified initial codes from the data by writing notes and memos about interesting content. We searched for patterns by looking for similarities and differences in the data to form the codes. Furthermore, to avoid excluding any content, we made efforts to include all of the data. In step three, all the codes were sorted by looking for similarities and differences and were grouped by potential themes. We tried all the potential themes in an initial “thematic map” of subthemes and themes. In the fourth step, we analyzed the relationships between the codes, themes, and different levels of themes. To check trustworthiness, the themes, codes, and subthemes were compared with each other and the whole. In step five, we defined and refined the characteristics of each theme, so that the subthemes gave structure to the main themes and captured the essence of each theme. To achieve credibility, we tried different names for the themes and worked to describe each theme clearly until a final thematic map was made (see Fig. 1). In the final step, we chose convincing extract examples and made a final analysis with feedback from the aim to confirm the results. Quotations are presented to illustrate the findings, which have been translated from Swedish to English by an authorized translator.

## Results

We asked the stakeholders how conditions for a sense of security could be created during the evenings and nights among older people receiving home health care. Additionally, due to the design of the research, we learned and reached a consensus together with the stakeholders. Therefore, there were no prominent differences between the stakeholders’ statements, as all the different groups agreed on the general concepts. However, the older persons and the NAs focused more on their interactions and how the environment interplays with the older individuals’ habits and routines. Relatives talked more about the older persons’ situations and what it was like to grow old. They saw a need of extra night supervision from the nursing staff. The RNs focused somewhat more on self-determination and creating opportunities for participation by the older persons. The nursing staff and managers also had more discussions on how to prevent falls.

Five subthemes were developed from the data, which give structure to the two main themes, To confirm the self-image and To create interaction in a sheltered place. The two themes are interdependent conditions. The person’s self-image must be confirmed to create interaction in a sheltered place, and through the interaction, the self-image is confirmed (see Fig. 1).

### To confirm the self-image

This main theme can be characterized as the home being seen as a reflection of the person’s identity. NAs and RNs helped the person with habits and routines in the manner that the person wished. Furthermore, they sought to maintain the person’s self**-**determination. Altogether, this helped to confirm the person’s self-image, which created conditions for the older person’s sense of security.

### To see the home as a reflection of the person’s identity

We learned together with the stakeholders that seeing the home as a reflection of the person’s identity created conditions for the older person’s sense of security. In this subtheme, among the conditions were the design of the home, the aesthetics, and its location. Another condition was that a familiar member of the staff could facilitate the older person’s ability to stay in place. While providing continuity, the caregiver helped the person to carry out their habits and routines, taking guidance from the older person to become their “hands and feet” in daily life.

We learned that the design of the home, the furniture, and the location of the home were all-important for the person to be as they always had been. The older people showed their homes to the researcher during the evening observations spontaneously and with pride. Some had created their homes elegantly and others more functionally. They showed photos and shared their memories. Several of the older persons lived in the home they had lived in for decades, in an area that was familiar, but where others had moved away because they lacked a sense of security. The community outside could give them a sense of insecurity, and there were those who did not go out in the evenings, even if they could. Older person: “*I don’t know what goes on outside the house.*” Even relatives and nursing staff reported that the community could create a sense of insecurity. They kept a watchful eye and warned the older persons of relevant dangers and told them if they saw something unusual.

We also learned from the stakeholders that the living environment was important to the older persons’ sense of security. Relative: “*It is important to live where it is beautiful.*” Observations revealed that health care aids, equipment, and other mobility aids were often left in plain sight. When we asked the older persons about this, they remarked that they perceived these things as intrusions in their homes that increased their sense of insecurity. However, they expressed ambivalence about these items, since the aids were also recognized as lifelines that contributed to a sense of security.

The NAs also stated that the older persons should live in a familiar environment that they were used to. They also told us that they had become a part of the older persons’ sense of security, where the older person guided them. NA: “*They show us how to be their hands and feet, and their contact with the outside world.*” Staff continuity was important in creating the conditions for a sense of security for both the older persons and the nurses that is, meeting the same person, a person they knew well. Obstacles to this were nursing staff stress and staffing problems that could lead to lack of continuity.

The older persons found it was a struggle to maintain habits and routines, that is, walking and moving, dressing, or going to the bathroom, because “their bodies did not obey them.” Pain could also be an obstacle to their mobility. Older person: “*I can’t put on my shoes, and that’s irritating. In the winter, someone has to help me dress. I don’t manage it myself, I have pain; it gives me a sense of insecurity.*” The NAs remarked how they saw the older persons’ sense of insecurity. NA: “*People can become insecure if they can’t manage going to the bathroom by themselves.*” According to the relatives, the ability to move, to get out, and to feel independent is conducive to a sense of security. Since the older persons had limited mobility, the stakeholders reported that the older persons needed assistance in their struggle to maintain their habits and routines.

The older persons said that the nursing staff should see what is needed and perform the tasks in the same manner as they would. Older person: “*Yes, they should see what needs to be done, and be precise. Most put the things by the sink away. It is second nature for them. The nursing staff here is outstanding*.” It became important for the NAs to get to know the older persons, to understand and learn their habits and routines, so they could learn how to perform them as the older persons had done previously. The NAs tried to adapt to the older persons’ limitations in different ways. Additionally, the nursing staff told us that the older people could have advanced nursing care in their homes, which could create continuity and a sense of security, since the home represents the person. Some NAs explained that, for the older persons, just knowing they could stay in place until their death created a sense of security.

### To maintain self-determination

We learned together with the stakeholders that the ability to maintain self-determination created conditions for the older people’s sense of security. In this subtheme, one condition was to be able to rely on themselves and to still be someone to count on. Among the other conditions were the ability to choose who would provide their care, and to participate in decision-making. The older people’s self-determination should be strengthened, and they should not have to adapt. However, sometimes the nursing staff was unable to meet the older people’s needs.

Further, we learned together with the stakeholders that conditional to a sense of security for older people was being able to rely on themselves. They knew what home health care they wanted and how it should be given. The older persons dared to complain about their care. Several of the older persons told us that they called directly to the manager and expressed their views, which gave them a sense of security. Older person: “*A person can complain, and I know I can complain if there is something. It is reassuring to know that it’s okay.*”

The observations revealed that more personal care was performed during late evenings and at night. Some needed help going to the toilet or replacing incontinence aids, and several needed help with their personal hygiene. Such care made it even more important for the older persons to be able to choose who should give the care. Some female older persons did not want male staff members to help them, as this was considered too intimate and created sense of insecurity.

The NAs pointed out that the older persons had the right to choose. However, this was considered a problem and a challenge in their work. NA: “*It is strange, because we need to bring men into the nursing profession and be 50–50, but the older women have the right to refuse the guys.*” There were male staff members who perceived it as difficult when someone refused to let them help with a shower. The managers agreed that men working in nursing could create conditions that lead to a sense of insecurity, especially for women from other cultures.

Older persons had experienced obstacles to their sense of security when they were excluded from decision-making. It was important for them to participate when decisions were being made. Older person: “*The health care system gives me a sense of insecurity. The doctor took away the pills without seeing me, without speaking to me or examining me.*” Older people explained that they wanted feedback regarding their health, medical follow-ups, and care planning. The other stakeholders reported that sometimes it was not always possible to meet the older person’s right to determine their care, which is an obstacle to their sense of security. NAs explained that if the older person is sleeping soundly, they are experiencing a sense of security, and therefore it is important to not awaken them unnecessarily. However, this poses a problem when analgesics are prescribed for 06:00, as that entails waking someone who has perhaps only slept a couple of hours and would not be able to fall back to sleep again. This causes insecurity, especially if the older person has to wait for the day staff. NA: “*The day shift wants the night shift to wake them up early and give them a pain killer so they can get them up. Then they are awake, and the day shift doesn’t come until several hours later. To be able to sleep is important, but the night is poorly respected.*”

To strengthen the older person’s participation in decision-making was important to the nursing staff. The RNs were aware of the importance of always informing the older persons about their care and encouraging them to participate in planning it. They were especially mindful to inform them about changes, and include them in all pertinent decision-making. RN: “*We must inform them, include them. Absolutely never do something over their heads that creates insecurity.*” The nursing staff emphasized that it was important to strengthen the older person’s self-determination. By doing so, the older person could be made stronger from within, which creates conditions that enhance participation and their sense of security. This inner strength could enable the older persons to make decisions and take command. NA: “*It is important to strengthen the person, so they can feel a sense of security from the inside.*”

It was also important that the older people not have to adapt to the nursing staff or the organization. They should be able to do as they had done before and remain the people they had always been. RN: “*If there is anything a person could do to help older persons to have a sense of security, it is allowing them to be the person they have been.*”

The RNs explained how the relatives could be helpful if they shared what they thought the older person’s needs were, since they knew the family member best. Actively inviting the relatives into discussions could be a way to get them to participate in their family member’s care. The relatives agreed that they and their family members could have a better sense of security if they were invited to participate in the care.

### To create interaction in a sheltered place

This main theme is characterized by the nursing staff interacting with the older people while they “undress the power.” Additionally, for a sense of security, the interaction should be in harmony with the environment in the home. Therefore, nursing staff need to create control and lifelines; they also have to ensure a good sleeping environment. Altogether, this could promote interactions in a sheltered place that result in older people having a sense of security.

### To undress the power

We learned together with the stakeholders that when the nursing staff undressed the power at the front door, they created conditions favorable to the older persons’ sense of security. In this subtheme, to act as an equal, to greet and relinquish the power before starting a conversation, and to be compliant and adapt to the older persons were among the conditions.

We learned that the older persons wanted the staff to see them as competent adults and not act superior or consider themselves better than them. Older person: “*You should be able to feel equal. If someone is arrogant, a person can have a sense of insecurity. It’s also not good to be ridiculous or treat us like small children; it fosters insecurity. It’s feeling like an equal that gives a sense of security.*” The staff should see the person and not just the illness. They should also act naturally, which means engaging in small talk and other things than just what is immediately apparent. O*lder person:* “*And be a little familiar with and able to talk a bit about the royalty and such. They should be able to talk about different things and what happens here.*”

In striving for equality, the NAs would relinquish their power when they greeted the older people at the front door. They rang the doorbell before they opened the door with the key. They loudly announced who they were as soon as they got inside the door. Before entering, they removed their jackets and took off their shoes or put on shoe covers. The NAs explained that it was also important not to show that they were stressed. NA: “*We’re not the ones with the power; it’s the older persons. It’s their home, and they have the power. It is important to take off your jacket. If you don’t, they’ll think you are in a hurry, and that’s something they absolutely shouldn’t think.*”

The NAs smiled while they chatted and gave compliments regarding, for example, someone’s hair or clothing. The observations also revealed that the nursing staff could talk while they performed the care or while sitting at the same level or lower than the older person. They engaged in small talk, including about recent news and what was happening in the community, and listened to the older person’s life story. The NAs stressed that it was important to act naturally and as an equal. NA: “*A sense of security is also about acting natural, so that they feel things are as usual. That the staff isn’t full of themselves or above them.*”

The NAs explained that they had to be compliant and adapt to the older person and the person’s way of being and living. It was important to try to understand their situation. They considered it inappropriate to walk into someone’s home, and with their own values and views, tell the person what to do. The observations and conversations with the NAs clearly showed that they changed how they interacted with each individual they encountered. NA: “*You have to change your approach all the time. What you do and how you meet people is different from person to person. Everyone is different, and you have to adapt yourself to each person.*” The nursing staff could not refuse to make a home visit if it involved something they considered “difficult” or “unpleasant.” They had to adapt and solve each situation separately.

### To create control and lifelines

We learned together with the stakeholders that to create control and lifelines creates interactions between the older people and their environment, which are conditions for an older person’s sense of security. In this subtheme, the conditions were the need to have control over habits and routines, to be one step ahead before something happened, and to prevent falls by employing, for example, equipment aids and nursing measures.

We learned from the stakeholders that the older persons had difficulties with moving, seeing, and hearing, resulting in limited mobility, dependency, and a fear of falling; all of which were obstacles to their sense of security. A sense of insecurity could arise from a limited and monotonous life caused by these difficulties. Older person: “*I don’t see so well anymore and I am dependent upon my children and home health care. Not being able to manage what I used to be able to, gives me a sense of insecurity.*” Those with a hearing impairment found it difficult to participate in conversations. The relatives explained that to manage daily life and feel independent are conditions for a sense of security. Relative: “*It’s independence a person strives for. Not being a burden on someone else. When the body work, you can feel free.*” Therefore, measures that give older people control over their daily lives, habits, and routines need to be created.

The older persons told us that if something unforeseen occurred, such as an NA being delayed, it could disturb the everyday “rhythm” of their habits and routines*,* and a sense of insecurity could ensue. The NAs therefore used different strategies to be one step ahead. Making schedules and plans together with the older individual was one method used to stay one step ahead and to create control and conditions for security. NA: “*It’s our duty to promote security. It is part of the job. When we make our first visit, we can give structure to the evening. Perhaps we don’t take the hardest thing first; instead, make plans to do it later the next time we come, so we can prepare the person.*”

We also learned that many older people fell during the evenings and at night, and all the stakeholders commented on this fear of the older person falling. The older people were afraid of falling and could not trust themselves, which created a sense of insecurity. The consequences of falls could be fatal. RN: “*Many older people fall a lot and hurt themselves. Many fracture their hips. If they are debilitated and 98 years old, they don’t recover.*” Relatives wanted their family members to have extra supervision from nursing staff at night, when they were afraid that something would happen. Older people had fallen both indoors and outdoors, and some had even sustained hip fractures. Older person: “*My blood pressure dropped when I was here in the apartment and I fell. I broke my hip. Now I can only go with a walker or use a wheelchair. I don’t manage without something. Before I broke my hip, I used to do all my own shopping. You become quite fragile when you break bones and get older. What makes me insecure is the fear of falling again.*”

Stakeholders saw the aids as lifelines and a condition for a sense of security, while at the same time, older people saw them as intrusions in their homes. With the help of a walker, older people could ambulate and go for a walk. They could also sit on the walker when they washed themselves or prepared their food. A security alarm gave them direct contact with the staff. Incontinence aids could also give a sense of security, as they allowed them to live in a usual manner and sleep the entire night. Older person: “*Security means not having to get up in the middle of the night. I have an incontinence aid. It is reassuring that I can sneeze and laugh—that’s real security.*”

In an effort to prevent falls, the NAs would do “security checks.” Before leaving the older people’s homes, they would make a visual “survey,” scanning the room to see that everything was in order and that everything the older people needed was placed close to them. NA: “*Yes, and everything is nearby that should be nearby. And that everything works.*” The managers and RNs also stressed the importance of security checks and of taking the time to ask the older persons if they needed anything else. It was also important that the older persons dared to ask for more help if they needed it.

The RNs, NAs, and managers said that many of the older persons managed their activities of daily living rather well during the days, but their abilities declined during the evenings and nights. They explained that ways to prevent falls were to have staff “walk” with the older persons during the day, and to make sure they had proper nutrition and plenty of fluids. To aid in this, it was suggested that the alarm center telephone the older persons and remind them to do some of their exercises and drink an oral nutritional supplement or other fluids. The managers also suggested that occupational therapists could be helpful in preventing falls, if, in addition to assessing the status of the older persons’ activities of daily living for the daytime, they also assessed them for the evenings and nights, since these often differed. Manager: “*At night it can be more difficult to see and move than during the day.*”

### To create a good sleeping environment

We learned together with the stakeholders that to create a good sleeping environment was to create conditions that enhanced a sense of security. In this subtheme, the condition was to have help with the small details that promote good sleep, which enables the older persons to interact harmoniously with their sleeping environment. Other conditions were to have nightly monitoring and to create individualized sleeping environments.

We learned that it was important for the older person’s sense of security to be able to sleep well, and that they had different strategies for this. It appeared in the observations that some of the older people slept sitting up, while others slept with the radio or television on, and some slept with their pets in their beds. Older person: “*I go to bed in the evening and fall asleep to the music. I wake up in the morning when the night staff makes their last visit and turns off the music.*” Others did not fall asleep until into the morning. The stakeholders agreed that a good night’s sleep was important for the older persons. The NAs helped them with the small details that could help promote sleep and create harmonious interactions between the older persons, the nursing staff, and the environment. Examples of this were having a glass of water on the nightstand or having a lamp lit.

The stakeholders explained how the nightly monitoring checks contributed to the older people’s sense of security, and enabled them to sleep. Some waited for the night staff’s first visit and could not fall asleep until they arrived. When the NAs came the second time, the person might be sleeping soundly.

During the observations it could be seen how the nursing staff interacted with the older people to create individual sleeping environments. When the nursing staff entered the older people’s homes at night, they were careful to not wake them if they were sleeping. They moved quietly, with restrained body language, and with their backs smoothly rounded. The NAs went to the bed and bent over the older person, and if the person did not talk or move, they checked that the person was breathing*.* NA: “*Best of all is if they snore when they sleep, because then we know everything is fine.*” The NAs reported that they were attentive and flexible regarding the arrangement of the individual sleeping environment. The observations revealed that the NAs helped those who needed to turn in bed so they could rest well. They arranged pillows and blankets in different ways and raised or lowered the head of the bed. The goal was that the older person would be asleep when the staff came back, but if the person was still awake and wanted to talk, the NAs stayed with them for a while. NA: “*You have to get to know which ones will want to talk at night.*” The older persons could use a security alarm to call for help, if they did not feel comfortable in bed. Nursing staff were observed returning several times to help persons rest comfortably in the bed. The managers stressed that the NAs were to arrange the individual sleeping environment to create conditions that promoted a sense of security. It was therefore important that there was documentation on how the older people preferred to sleep.

## Discussion

Two main themes, with subthemes, emerged out of the resulting data. Main theme one, To confirm self-image, has two subthemes: To see the home as a reflection of the person’s identity, and To maintain self-determination. Main theme two, To create interactions in a sheltered place, has three subthemes: To undress the power, To create a sense of control and lifelines, and To promote a good sleeping environment. The themes interact and support each other in the following way: to see the home as a reflection of the person’s identity and to maintain self-determination could confirm the individual’s self-image. Nursing staff can create interactions in a sheltered place with the older person when they undress the power, create control and lifelines, and create a good sleeping environment (see Fig. 1). The themes and subthemes, that is, the overall results, are about the older person’s self-image and identity, which are confirmed and evolve from interactions with nursing staff and the environment in the older person’s home, in ordinary housing. This is in accordance with Mead’s theory on symbolic interactionism and the concepts of “I and Me” and the “generalized other.” To deepen the understanding of the results, Mead’s theory is used in this discussion. According to Mead, the individual’s self-image and identity evolve throughout life. Identity can be seen as a social product that is constantly constructed and transformed through interaction with others and society, where the interaction affects one’s self-image and identity [38–40]. Thus, identity and how one sees oneself develops throughout the life course. This idea of identity, interaction, and development has the same relevance in this study regarding the sense of security. Therefore, it becomes important to know how conditions for a sense of security are created by nursing staff in their interactions with older people in ordinary housing. Mead uses the terms “I” and “Me,” where the Me is the socialized aspect of the person, and the I is the active, individual aspect of the person. Mead [39] also uses the term “generalized other” as the link between I and Me that makes the social part of the individual and vice versa. The generalized other represents the attitude of a larger system, such as the community and/or the organization within, and in this study, home health care. Thus, within home health care, the attitudes about how something should be or how it should be performed are assumed from the generalized other [39], which can affect the interactions between the older people and the nursing staff.

### To confirm self-image

The older person’s home, together with its location and design, is important for confirming their self-image. The home is a reflection of a person’s identity, and it can be seen as an inner world to those who live there [38, 39]. It could provide protection from the outside world [41] and affect one’s health and well-being [42]. The home is a place where it is easier to be oneself, to maintain one’s self-identity [43, 44]. Its esthetics can be seen as a description of the self and the life story [38, 39]. Interactions with nursing staff made it possible for the older persons to stay in ordinary housing, in a place that offers them shelter and confirmation of “self” and self-image. Maintaining habits and routines is not just a task-oriented duty, but depending on how the actions are performed, can confirm the individual’s lifestyle and identity [45, 46]. In this study, the prerequisite for being able to maintain habits and routines and create a familiar context was that both the older persons and the NAs knew each other well, which required continuity. Continuity and security are in fact and in reality intertwined [47]. The staff also need to be well acquainted with the context to be able to understand themselves in their interactions with others, as a “Me” implies the ability to see themselves through the eyes of others and to interact in a meaningful way [38–40]. Time and continuity are important for a sense of security and for aging and staying in place [27–29].

For older adults to achieve a sense of security, maintaining self-determination is vital. This way of thinking coincides with Swedish laws and guidelines, which clearly state that everyone is entitled to a dignified life; people should be allowed to have influence over their lives and they should be allowed to participate in their care [26, 48, 49]. In agreement with Mead [38–40], this could facilitate the individual’s self-determination and help to create interactions that confirm and strengthen the older person’s self-esteem and identity.

### To create interaction in a sheltered place

In the interactions, the nursing staff undressed their power to create conditions conducive to a sense of security: they were compliant and adapted to the person they were meeting. They interacted via gestures, facial expressions, and small talk, interactions that are also important for the older person’s well-being [50–53], and in this case, for the conditions necessary for a sense of security in a sheltered place. The NAs were attentive to what the older persons were doing “now” and previously [52–54], that is, their life stories, which creates identity. According to Mead [38–40], they adopted the person’s perspective and embraced their situation and feelings.

It was important to have a sense of control and lifelines, since the older people’s bodies did not obey them. There is, according to Mead, a clear division of body and soul when the body cannot be trusted [52, 53]. Other research shows that good mobility and functional capacity give a sense of freedom and control [17], which is important for a sense of security [45, 46]. Having reduced mobility may cause difficulties interacting with the environment, managing habits and routines, and living as usual. Reduced mobility can also threaten one’s self-image and identity [38–53]. A breakdown in the personality and identity can also occur [38].

In the interactions, the NAs wanted to get to know the older person to learn what habits and routines the older person had. They were guided by the older person and performed the habits and routines with accuracy, striving to be one step ahead, which can promote conditions conducive to a sense of security [45, 46]. Some of the older persons had difficulties seeing and hearing, which could be a threat to their interaction with the environment and create a sense of insecurity*.* Falling can also be a threat to one’s identity [55].

The nursing staff did “security checks” before they left. These were ways of facilitating interaction with the environment, thereby preventing a division between the I and Me and maintaining the older persons’ identities. This can result in the older persons gaining control, which can promote conditions for a sense of security [45, 46].

Also important for a sense of security was a good sleeping environment. There is a significant association between insomnia and a person’s well-being and their physical and mental health [56]. It is therefore important to know the older persons’ sleeping habits [57]*.*

### Obstacles to creating conditions for a sense of security

Several interactions could present obstacles to older persons realizing their self-image and the interaction in the sheltered place and thereby their sense of security. At the same time as the older persons saw the health care aids and equipment as lifelines, they saw them as intruders in their home. It is known that such aids can compete with other things that represent the individuals’ identity [58]. Others have also reported that they are ashamed of using aids [59]. However, having conflicting feelings and beliefs about the same thing can be seen as two sides of the same coin [60]; accepting this can be a way of dealing with daily life [61]. Occurrences such as the NAs having to awaken older persons to give them analgesics in preparation for the day staff coming to get them up are also obstacles. To awaken someone due to the needs of the organization, can, according to Mead, objectify the person and create an interaction that demeans the person’s identity [52, 53]. Furthermore, the older people were not always allowed to make decisions or choose who could help them with their personal hygiene. It has been reported that older persons with home health care could wait a long time to have help showering and were limited in deciding their own spontaneous choice of activities [62]. When a person is not asked or allowed to choose, their self-image and sense of security can be threatened. The health care organization considered the right of the older person to decide in the interactions, but constraints imposed by the organization could hinder this. The norms and values of the self, that is, of the older person, are in conflict with the norms and values represented by the generalized others, that is, the elderly care organization [38, 39]. These norms and values can lead to the view that older people are fragile and lack adequate knowledge, which results in decisions being made over their heads [10].

## Method discussion

Using the PAAR design to describe how conditions for a sense of security can be created during the evenings and nights for older persons turned out to offer great opportunities. The repeated cycles of collecting data and taking them back again to the stakeholders is a strength, and it brings trustworthiness to the study, since we could ask the stakeholders about things that were unclear as well as deepen our knowledge [63]. Trustworthiness has also been achieved through a triangulation of data that included interviews, observations, and informal conversations. Furthermore, the final interpretation of themes has been considered and confirmed by all authors. Throughout the entire research project, we used openness and strived to be aware of and critical of our own preconceptions and to learn from the stakeholders to achieve validity and quality [64].

However, some other questions must be discussed. First, a weakness is that we could have gone back once more when the results were completed, but the study was time consuming in its nature and it was a challenge to reach consensus. The stakeholders’ role as co-researchers could then have been strengthened. According to Bindels et al. [65], there is a risk of researchers underestimating the involvement of older persons as co-researchers. Second, another weakness in the study is that only a few relatives participated. However, it is well known that relatives who also are caregivers are often exhausted [21, 22, 66]. Third, the stakeholders did not always separate the days and nights in their reflections, which is a weakness. On the other hand, it could be difficult to separate days, evenings, and nights when studying the sense of security, since it is an existential concept influenced by the person’s whole life. Finally, the majority of the stakeholders and the researchers were female, which could be a weakness affecting the transferability. We could have learned more with both female and male stakeholders. However, in Sweden’s municipal care sector, the majority of the employees, the older persons, and the older persons’ relatives are female [67].

## Conclusion

We learned together with the stakeholders that living in a familiar environment is a condition that promotes older people’s sense of security. That the nursing staff and the older persons know each other, so that there is continuity and so that, for example, habits and routines can be maintained and performed as if the older persons performed them themselves, is another condition. Self-determination is a vital condition to confirm the person’s self-image. Other conditions are equality, with the undressing of the power, the creation of control, and being one step ahead. “Security checks” and aids such as walkers and security alarms are seen as lifelines. Further conditions are the prevention of falls and an individualized good sleeping environment. These conditions create interaction between the staff, the older person, and the home environment so the person can live in a sheltered place. Through these interactions the person’s self-image is confirmed. Older people’s self-determination should be honored. They should not being excluded from decision-making, but rather helped to overcome the structural and institutional obstacles to remaining independent and feeling secure. We need to ask them if the conditions are sufficient and their sense of security is great enough to allow them to continue living in their ordinary homes.

## Data Availability

The data compiled during the study are available, in Swedish, from the corresponding author upon request.

## Abbreviations

NA: Nurse assistant
RN: Registered nurse
